# Multi-omics analysis revealed the role of CCT2 in the induction of autophagy in Alzheimer’s disease

**DOI:** 10.3389/fgene.2022.967730

**Published:** 2023-01-10

**Authors:** Xueting Ma, Yuxin Feng, Xiangyu Quan, Bingyu Geng, Guodong Li, Xueqi Fu, Linlin Zeng

**Affiliations:** ^1^ Edmond H. Fischer Signal Transduction laboratory, School of Life Sciences, Jilin University, Changchun, China; ^2^ Department of General Surgery, The Second Hospital of Jilin University, Changchun, China

**Keywords:** alzheimer’s disease, autophagy, CCT2, microRNA, logistic model

## Abstract

*Chaperonin containing TCP1 subunit 2* (*CCT2*) is essential in various neurodegenerative diseases, albeit its role in the pathogenesis of Alzheimer’s disease (AD) remains elusive. This study aimed to evaluate the role of *CCT2* in Alzheimer’s disease. First, bioinformatics database analysis revealed that *CCT2* was significantly downregulated in patients with Alzheimer’s disease and associated with autophagic clearance of β-amyloid. The 789 differentially expressed genes overlapped in AD-group and *CCT2*-low/high group, and the *CCT2*-high-associated genes screened by Pearson coefficients were enriched in protein folding, autophagy, and messenger RNA stability regulation pathways. These results suggest that *CCT2* is significantly and positively associated with multiple pathways linked to autophagy and negatively associated with neuronal death. The logistic prediction model with 13 key genes, such as *CCT2*, screened in this study better predicts Alzheimer’s disease occurrence (AUC = 0.9671) and is a favorable candidate for predicting potential biological targets of Alzheimer’s disease. Additionally, this study predicts reciprocal micro RNAs and small molecule drugs for hub genes. Our findings suggest that low *CCT2* expression may be responsible for the autophagy suppression in Alzheimer’s disease, providing an accurate explanation for its pathogenesis and new targets and small molecule inhibitors for its treatment.

## Introduction

Alzheimer’s disease (AD) is a neurodegenerative disease responsible for 60–80% of dementia cases, which is characterized by memory loss and reduced cognitive function (Liu et al., 2020). This report indicates that neuronal fibrous tangles caused by Tau hyperphosphorylation in neurons, sedimentation of amyloid beta (Aβ) plaques (Ma et al., 2022), apoptosis of numerous neurons, and loss of neural synapses all contribute to AD. Drugs approved by the FDA for AD are designed to improve the quality of life of patients with the disease albeit may not play an effective therapeutic role in the treatment of AD (2020 AD facts and figures, 2020). AD-related therapeutic drugs based on Aβ starch spot and Tau protein have not made significant progress (Kopeikina et al., 2011); thus, the development of the specific pathogenesis of AD requires further research and exploration. It has been reported that the *chaperonin containing TCP1 subunit 2* (*CCT2*) is poorly expressed in AD; however, the relationship between *CCT2* and AD remains elusive (Yuan et al., 2019), implying that there is some link between *CCT2* gene expression and the occurrence of AD.

Aggrephagy, a process in which autophagy selectively degrades protein aggregates, is important for removing intracellular toxic protein aggregates and is a key target for the treatment of aggregate-related diseases such as neurodegenerative diseases. Several studies have reported that autophagy deficiency occurs in the early stages of AD (Vaillant-Beuchot L et al., 2021; Roca-Agujetas V et al., 2021). Autophagy is important in the production and metabolism of Aβ, and its dysfunction may contribute to the progression of AD (Li et al., 2017). Traditional ubiquitin-binding receptors (*P62*, *NBR1*, and *TAX1BP1*) can mediate aggrephagy and other types of ubiquitin-related selective autophagy (Zellner S et al., 2021). The novel ubiquitin-binding receptor, *CCT2*, promotes autophagic clearance of various toxic protein aggregates associated with neurodegenerative diseases (Zhang and Klionsky., 2022). Similar to the conventional ubiquitin-binding receptors, *CCT2* binds to LC3 and protein aggregates. *CCT2* binds protein aggregates in a ubiquitin-independent manner through its apical domain, laying the groundwork for *CCT2*-specific aggregate recognition. Research has indicated that conventional autophagy receptors degrade liquid aggregates whereas *CCT2* degrades solid aggregates (Ma et al., 2022). Consequently, *CCT2* is more likely than autophagy receptors to function and become an AD drug target in pathological states. *CCT2* mediates aggrephagy as a monomer, exposing the VLIR domain of the binding site to LC3. The presence of aggregates inhibits the formation of the Chaperonin complex, thus, releasing more *CCT2* monomers to promote aggregate clearance (Khaminets et al., 2016; Johansen and Lamark, 2020; Gatica et al., 2018). Full-length tau protein has been reported to preferentially be degraded by macrophage whereas caspase-cleaved tau, tauΔC, which is more likely than natural proteins to aggregate and cause neurotoxicity, is preferentially degraded by autophagy and can turnover faster than the full-length tau. Thus, the autophagy degradation pathway is important in inhibiting the formation of pathological manifestations of AD and has the potential to be a novel target for its treatment (Zare-Shahabadi et al., 2015).

Therefore, this study aimed to investigate the changes in the expression level of *CCT2* in patients with AD and its possible pathway involved in autophagy and predict the possible micro RNA targets. Our study may help researchers investigate how *CCT2* affects AD *via* autophagy, contributing to the understanding of disease causes, mechanisms, and treatments.

## Materials and methods

### Data acquisition

All the datasets used in this study were obtained from the Gene Expression Omnibus database (https://www.ncbi.nlm.nih.gov/geo/) (Barrett et al., 2013). The AD transcriptome datasets screened from the database included brain tissue sequencing samples, GSE33000, GSE44768, GSE44770, and GSE44771, based on the GPL 4372 platform, peripheral blood samples of patients with AD, GSE140829, based on GPL5988 platform, and serum microRNA (miRNA) sequencing samples, GSE120584, based on the GPL21263 platform. GSE33000, which included 310 patients with AD and 157 controls, was used to explore the potential role of *CCT2* in AD. GSE44768, GSE44770, and GSE44771 were obtained from the cerebellum, frontal cortex, and visual cortex, respectively, and included 129 patients with AD and 101 controls. GSE140829 included 204 patients with AD and 249 controls to validate the model and explore *CCT2* expression in different tissues. GSE120584 included 1,021 patients with AD and 288 controls and was used to probe the possible messenger RNA (mRNA)-miRNA interaction networks. Component differences were observed using principal component analysis (PCA) plots drawn by the FactoMineR and factoextra packages. The data in GSE33000 was normalized using the normalizeBetweenArrays function in the Limma package (Ritchie et al., 2015), and the first group was retained for duplicated genes in the sequencing data.

### Screening of differential genes (DEGs) and associated genes

LmFit, eBayes from the limma package, and the topTable function were used to identify differentially expressed genes DEGs between AD-con and *CCT2*-low/high expression groups. According to the false discovery rate (FDR), *p* ≤ 0.05 was statistically significant, and log2fold change (FC) was used to comprehensively analyze the upregulated and downregulated genes.

For the AD-con group, we selected the first 30% genes with larger |logFC| under the *p* ≤ 0.05 condition as the DEGs. Further, we divided all patients with AD into high-and low-expression groups based on the median of *CCT2*, and under the *p* ≤ 0.05 condition, |logFC| the larger top 10% genes were selected as DEGs in the *CCT2*-low/high group. DEGs were intersected between AD-con and *CCT2*-low/high groups for further analysis.

Cor function was used for the raw data, and Pearson correlation analysis was performed between *CCT2* and other genes. If *p*-value was ≤0.05 and the gene was positively associated with *CCT2*, it was selected as the related gene.

### Functional enrichment analysis

*CCT2* and 460 genes with the strongest positive correlation with *CCT2* were uploaded to the online Fdatabase—Database for Annotation, Visualization, and Integrated Discovery, 2021 (Sherman et al., 2022; Huang et al., 2009)—for analysis. The official gene symbol was selected as the identifier, and the species was *Homo sapiens*. This was followed by Gene Ontology (GO) and Kyoto Encyclopedia of Genes and Genomes (KEGG) pathway enrichment analysis. The top eight pathway are displayed in ascending order of *p*-value (*p* ≤ 0.05).

### Gene set enrichment analysis (GSEA)

The differential expression analysis results of the limma package were analyzed by GSEA using the gseKEGG and gseGO functions of the clusterProfiler package in R (Wu et al., 2021), and biological process (BP) GO terms and KEGG pathways that may be related to AD and CCT2 expression were explored. *p* ≤ 0.05 and |NES| >1 indicated significant differences.

### Gene set variation analysis (GSVA)

Gene sets related to autophagy and protein folding were obtained from the GSEA website (http://www.gsea-msigdb.org/gsea/index.jsp) (Subramanian et al., 2005; Mootha et al., 2003). Before standardization, the GSVA package in R was used to calculate the functional enrichment scores of all AD groups in the GSE33000 dataset, and the parameters were set as default (Hänzelmann et al., 2013). Results were visualized by drawing heatmaps using the pheatmap package in R, and Pearson correlation analysis was used to determine the correlation between *CCT2* and autophagy and protein folding processes. Further, the top ten genes with the strongest positive and negative correlation with *CCT2* were drawn to exhibit their correlation with *CCT2* using data from the HADb database (http://www.autophagy.lu/index.Autopophagy-related gene sets of html) (Moussay et al., 2011) and circos package (Krzywinski et al., 2009). Relevant gene sets from different stages of autophagy were selected for GSVA analysis; Pearson correlation analysis was used to calculate its correlation coefficient; the corrgram package was used to construct matrix plots.

### Construction of the protein-protein interaction (PPI) network and identification of the hub genes

The DEGs from the AD-con groups intersected with the *CCT2* low-high groups, and 295 upregulated and 494 downregulated genes were removed as co-DEGs and uploaded to the online database (STRING version 11.0, https://cn.string-db.org/) (Szklarczyk et al., 2021) to predict the PPI network, with the default parameters. The PPI interaction network was further drawn using Cytoscape, and 36 hub genes associated with *CCT2* were removed using the MCODE plugin.

### Logistic model construction and receiver operating characteristic (ROC) curve analysis

The least absolute shrinkage and selection operator (LASSO) is a compression estimation method that has a strong factor screening ability (Tibshirani,1997; Zou et al., 2019). The hub genes were intersected using Pearson’s analysis results (|r|≥0.65, *p* ≤0.05) to obtain 26 genes, and the expression profiles of these genes were used to construct the LASSO model, with 13 genes whose regression coefficient was not zero. These genes were used to construct a logistic regression model using the glmnet package. This model had the following formula: index = EXGene1×Coef1 + EXGene2×Coef2 + EXGene3×Coef3+…… (Coef was the regression coefficient, derived from the logistic regression (Domínguez-Almendros et al., 2011); EXGene was the gene expression level).

Further, data from the GSE33000 dataset were randomly assigned to the test set (30%) and validated with those of the GSE44768, GSE44770, GSE44771, and GSE140829 datasets, and the ROC curve was drawn using the pROC package.

### *CCT2* expression and single-cell correlation analysis in different brain tissues

Using the online database, AlzDate (http://www.alzdata.org/) (Xu et al., 2018; Zhang et al., 2019) and the Single Cell Expression tool, *CCT2* expression in single cells was obtained. Using the Differential Expression tool, the differential expression of *CCT2* in multiple databases was obtained.

### MicroRNA-mRNA interaction network analysis

MicroRNA is a type of single-stranded RNA molecule that is encoded by endogenous genes and binds to mRNA inside cells to inhibit protein translation. Exploring the interaction between miRNA and its target genes can provide a reference for investigating the disease causes and therapeutic methods. Databases for predicting gene-miRNA interactions include MiRDB, miRWalk, RNA22, and RNAInter (Chen and Wang, 2020; Dweep et al., 2011; Miranda et al., 2006; Lin et al., 2020). They were used to predict the miRNA interactions with hub genes, and the results were cross-checked to improve prediction accuracy. Simultaneously, serum miRNA sequencing samples (GSE120584) were analyzed for differential expression using the limma package, and miRNAs with *p* ≤ 0.05 were considered differentially expressed. Additionally, the differentially expressed miRNAs that interacted with hub genes were visualized using the Cytoscape software.

#### Related drug prediction

Drug development has always prioritized research on drugs for AD. Numerous effective drugs are ineffective in AD treatment as they cannot cross the blood-brain barrier (BBB) whereas small-molecule drugs have natural advantages in crossing the BBB.

*CCT2* has been reported to be used as a target of small-molecule drugs in the treatment of neurodegenerative diseases. Consequently, the prediction of *CCT2*-related DEGs serves as a reference for AD therapy. The Drug Signatures database (DSigDB) on the Enrichr website was used in this study to identify relevant targeted drugs for DEGs (Chen EY et al., 2013; Kuleshov et al.,., 2016; Xie Z et al., 2021). The results were reviewed and displayed (Kuleshov et al., 2016).

## Results

### Identification of the DEGs in AD

**FIGURE 1:**
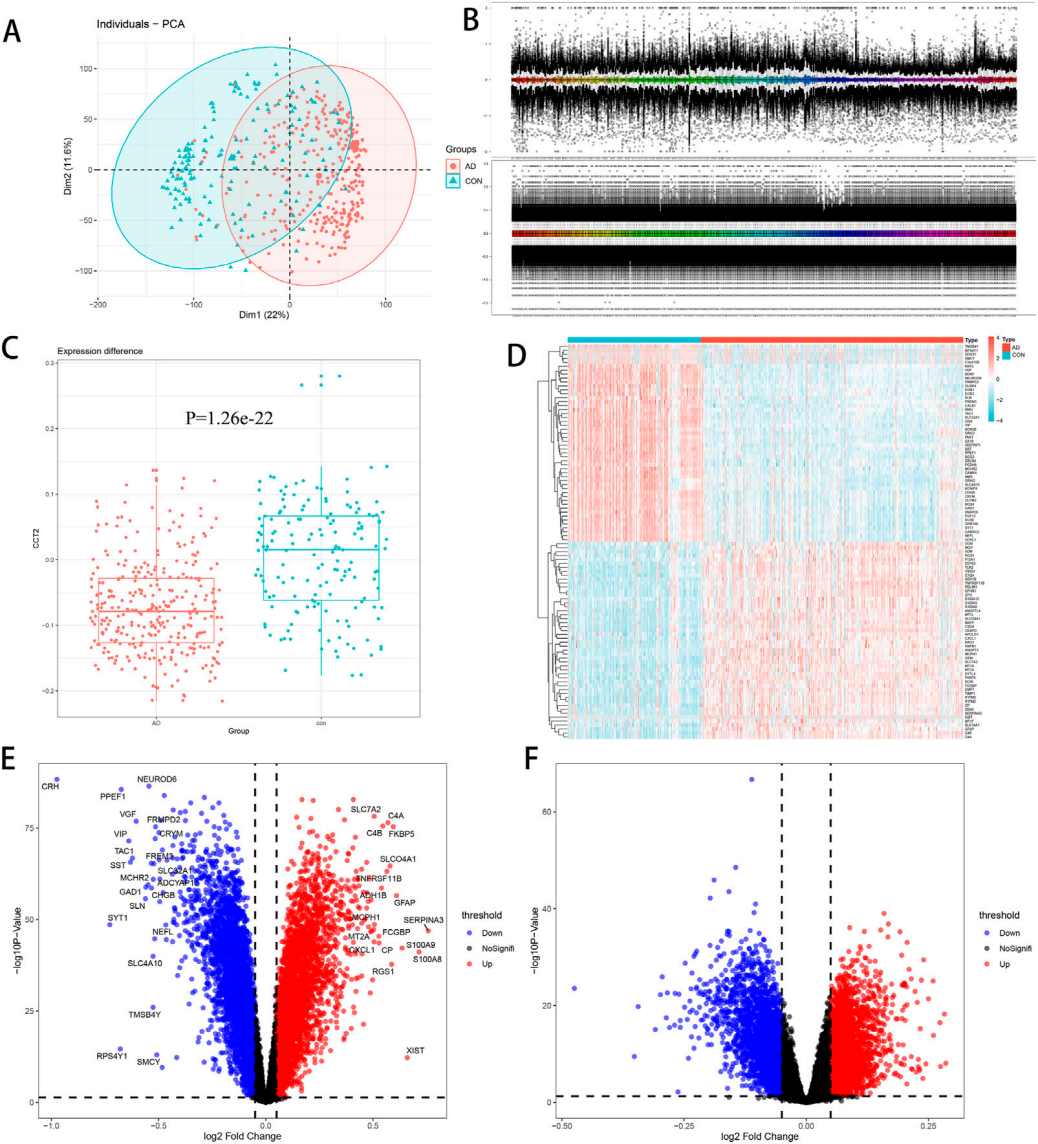
Differential expression analysis. **(A)** Principal component analysis (PCA) plot demonstrating differences among groups, with Alzheimer’s disease (AD) in red and con in green. **(B)** Boxchart before and after standardization—the upper figure is before standardization; the following figure is after standardization. **(C)** Chaperonin containing TCP1 subunit 2 (CCT2) was downregulated in AD (*p* = 1.26e-22, logFC = -0.07632). **(D)** Heatmap of the top 50 upregulated and downregulated genes between AD and control. **(E)** Volcano plot of AD-con, with upregulated genes in red and downregulated genes in blue. **(F)** Volcano plot of CCT2-low/high, with upregulated genes in red and downregulated genes in blue.

To investigate the differences in gene transcriptome between AD and normal controls, we conducted the following analysis. First, the PCA chart demonstrated that there are significant differences between AD and con groups in GSE33000, allowing for subsequent analysis (Figure 1A). Boxchart displayed standardized data, eliminating intra-group differences (Figure 1B). Second, we observed that *CCT2* was significantly downregulated in AD, *p* = -1.26e-22 and logFC = -0.07632 (Figure 1C), which suggested that the low *CCT2* expression is associated with AD. There were 4,381 DEGs in AD, with 2,152 upregulated and 2,229 downregulated genes (Figure 1E), among which the top 50 upregulated and 50 downregulated genes are indicated in the heatmap (Figure 1D). However, the *CCT2*-low group had 1,273 DEGs compared with the *CCT2*-high group, of which 561 were upregulated and 712 were downregulated (Figure 1F). A total of 789 genes were either upregulated or downregulated in the AD-con and *CCT2* low-high groups, which may be associated with both *CCT2* expression and AD.

### *CCT2* downregulation was linked to protein misfolding and neurodegenerative diseases

**FIGURE 2:**
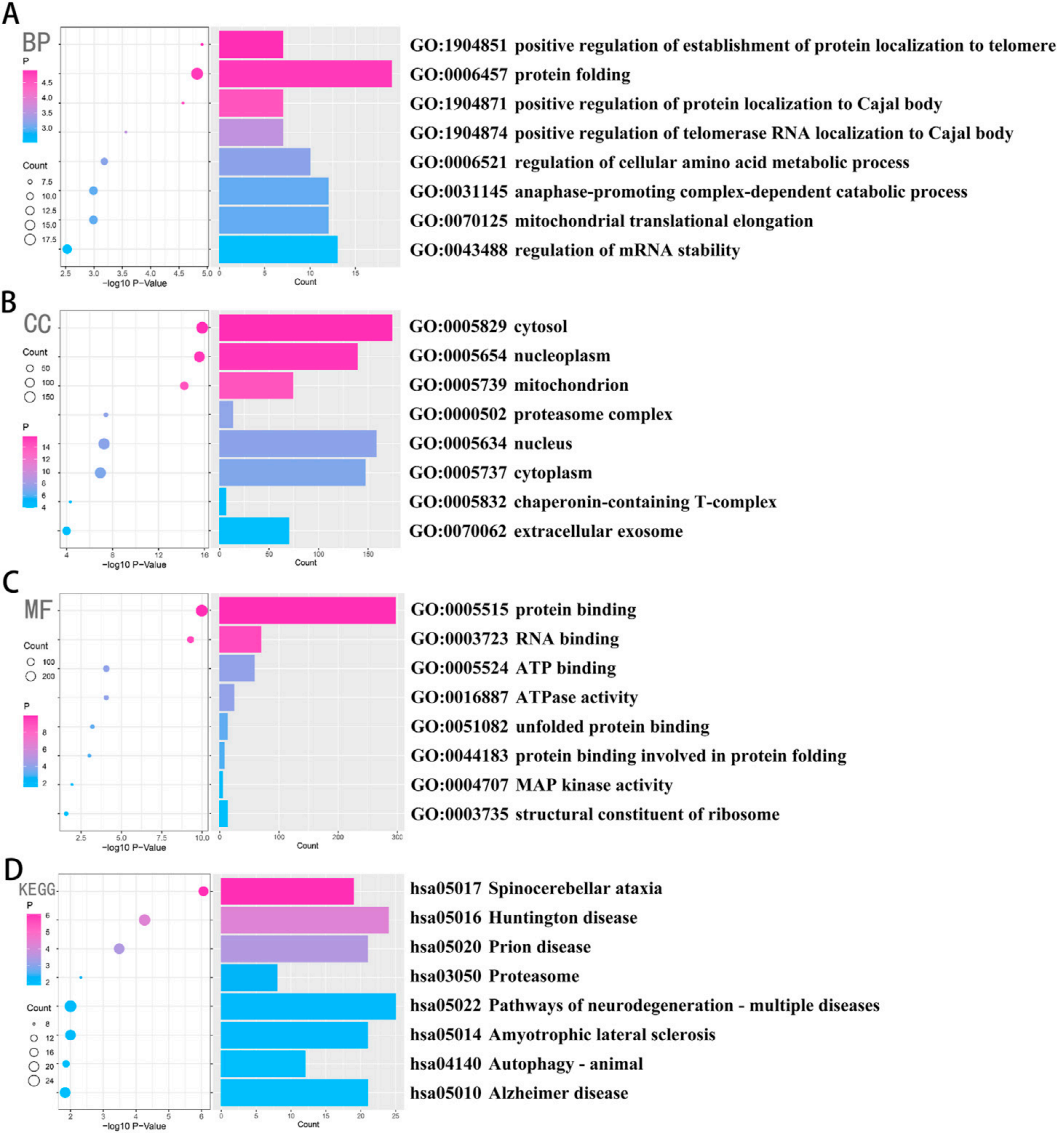
Chaperonin containing TCP1 subunit 2 (CCT2) was closely related to the process of β-amyloid formation and clearance in AD. **(A–C)** Biological processes (BP), cellular components (CC), and molecular functions (MF) were enriched in gene sets that were positively associated with CCT2 in Pearson’s test, whose credibility gradually increases from blue to red, and the size of the circle exhibits the number of genes contained in the corresponding pathway. **(D)** The signaling pathways (Kyoto Encyclopedia of Genes and Genomes (KEGG)) were enriched in the gene set that was positively associated with CCT2 in Pearson’s test, whose credibility gradually increased from blue to red, and the size of the circle exhibits the number of genes contained in the corresponding pathway.

Four hundred and sixty genes with the strongest positive correlation with *CCT2* were selected using Pearson correlation analysis to explore the relevant biological functions of *CCT2*. GO and KEGG analyses were performed according to the above-mentioned gene sets. Genes associated with *CCT2* in biological processes (BP) are primarily enriched in protein folding pathways, regulation of telomere protein localization related to the Cajal body, and regulation of mRNA stability (Figure 2A). Additionally, the most relevant cellular components (CC) of *CCT2* and its related genes included cell cytoplasm and T complex proteins (Figure 2B) and were related to exosomes (Figure 2C), whose molecular function (MF) was protein binding and folding, RNA binding, and ribosome composition. Moreover, the most related signaling pathway (KEGG) was mainly associated with various neurodegenerative diseases, including AD, and autophagy (Figure 2D). These findings indicated that downregulating *CCT2* in patients with AD may be significant for snRNP formation, mRNA splicing, protein folding, and clearance of misfolded proteins by autophagy. Thus, *CCT2* was associated with the production and clearance of amyloid proteins, and a possible cause of AD was *CCT2* downregulation.

### *CCT2* positively regulates the occurrence of multiple autophagy and reduces neuronal death

**FIGURE 3:**
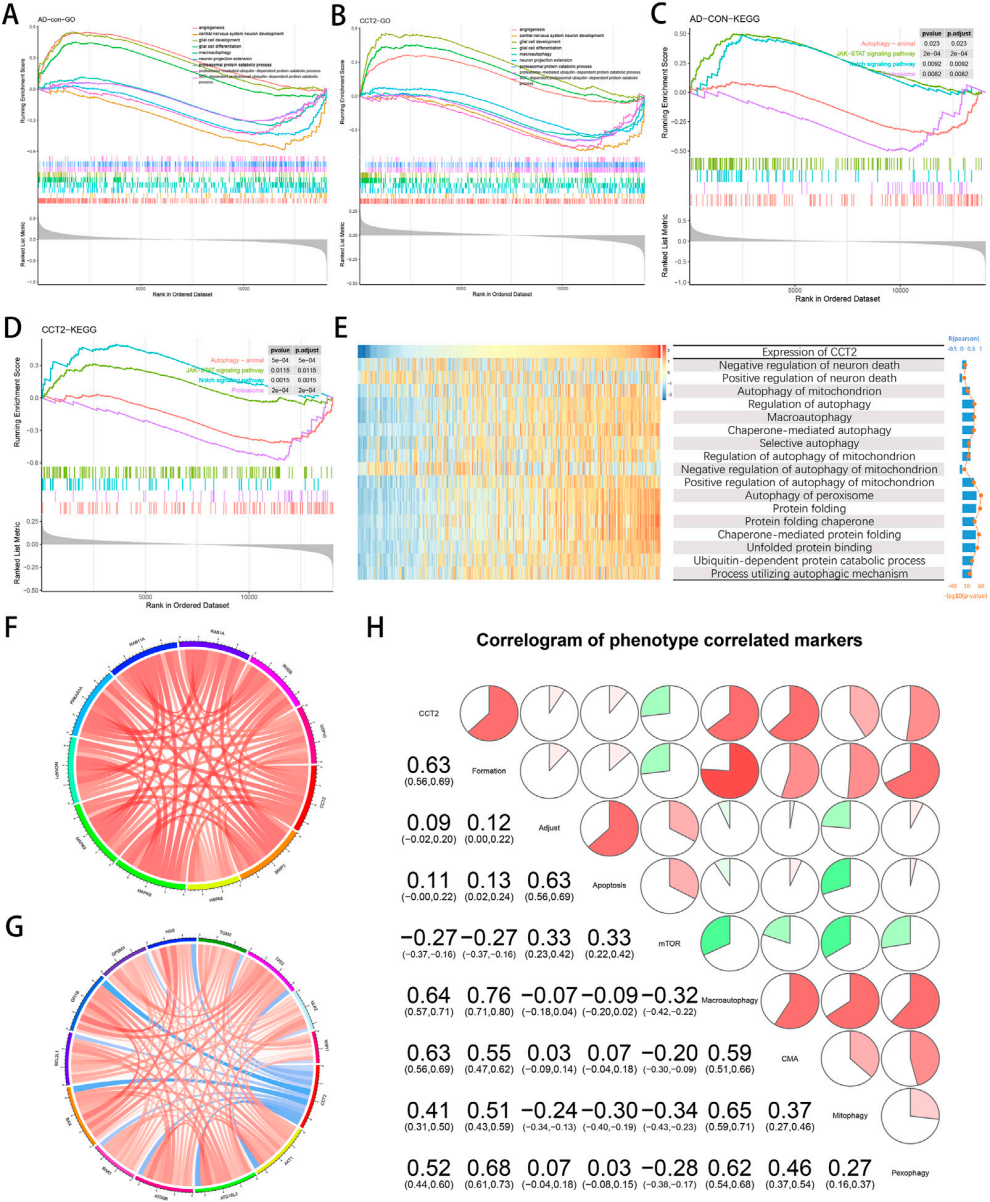
Correlation of Chaperonin containing TCP1 subunit 2 (CCT2) expression with autophagy and other gene sets. **(A)** Biological processes (BP) of Alzheimer’s disease (AD) enrichment. **(B)** BP of CCT2-low enrichment. **(C)** The Kyoto Encyclopedia of Genes and Genomes (KEGG) pathways enriched in AD. **(D)** The KEGG pathway enriched in CCT2-low. **(E)** Heatmaps display the enrichment scores for CCT2 expression and related pathways in GSE33000; samples are arranged in CCT2 ascending order, and bar and line plots on the right indicate the analyzed R and *p*-values. **(F)** Autophagy-related genes positively associated with CCT2, as indicated in red. **(G)** Autophagy-related genes negatively associated with CCT2, as indicated in blue. **(H)** Correlation of CCT2 with autophagy-related gene sets, the correlation coefficient is presented in the lower left and simultaneously in the upper right, red for positive correlation and green for the negative correlation.

The transcriptomic data was analyzed using GSEA and GSVA. In GSEA, the Janus kinase (JAK)-signal transducer and activator of transcription (STAT) signaling pathway and the Notch signaling pathway were significantly enriched in the AD group compared to the con group; however, there was a contrasting observation in proteasome and animal autophagy (Figure 3A). Meanwhile, there was a similar trend in the *CCT2*-low/high group (Figure 3B). Additionally, compared with the con group, glial cell development and differentiation and angiogenesis-related pathways were significantly enriched in AD, opposing the observation in protein catabolism and neuronal development (Figure 3C), with a similar trend in the *CCT2*-low/high group (Figure 3D). This suggests that the downregulation of *CCT2* may be a cause of AD. It has been proven that the accumulation of amyloid protein can affect the production of angiogenic factors (Skaaraas et al., 2021).

In AD progression, *CCT2* may alleviate amyloid aggregation by promoting normal protein folding and autophagy. Thus, the method used by the study was GSVA for calculating the enrichment score of *CCT2* expression levels for several pathway and BP. These include neuronal death regulation, mitochondrial autophagy, chaperone-mediated autophagy, peroxisomal autophagy, and other related pathway. BP such as protein folding and its decomposition. The enrichment score indicated that *CCT2* was positively correlated with most autophagy-related BP and protein folding; however, it was reversed in the negative regulation of neuronal death and the negative regulation of mitochondrial autophagy. (Figure 3E). These results indicate that high *CCT2* expression can inhibit neuronal death while enhancing mitophagy, which is considered to be the target of AD treatment (Xie et al., 2022).

Since *CCT2* was observed to be involved in AD, we investigated its relationship with the expression levels of some important autophagy-related genes. The results indicated that *CCT2* expression was positively correlated with *MAPK8*, *HSPA8*, NCKAP1, *RAB11A*, and *RAB1A* (Figure 3F), and negatively associated with *BAX*, *MAPK3*, ITGB4, *ATG16L2*, and ERBB2 (Figure 3G). Using the Pearson matrix diagram (Figure 3H), high *CCT2* expression revealed a significant and positive correlation with autophagy formation, macroautophagy, and autophagy mediated by molecular chaperones and a negative correlation with the mTOR pathway. This validates that *CCT2* downregulation affects normal autophagy for clearing Tau and Aβ, thus, causing AD.

### The logistic model was constructed for AD prediction

**FIGURE 4:**
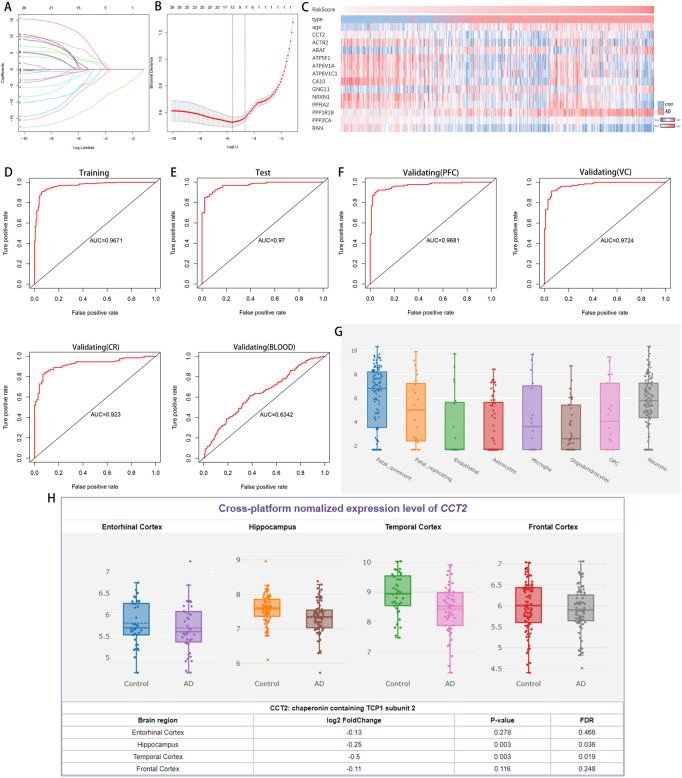
A logistic model for identifying Alzheimer’s disease (AD) and the expression of Chaperonin containing TCP1 subunit 2 (CCT2) in different tissues and cells. **(A–B)** Least absolute shrinkage and selection operator (LASSO) model. **(C)** Heatmap demonstrates changes in category, age, and gene expression as predictive scores rise. **(D)** Receiver operating characteristic (ROC) curve analysis of the training set (GSE33000). **(E)** ROC curve analysis of the validation set (GSE33000). **(F)** The ROC curve analysis of the external validation set (GSE44768, GSE44770, GSE44771, and GSE140829). **(G)** CCT2 expression observed in various cells of the brain. **(H)** CCT2 was significantly downregulated in various brain regions of patients with AD.

Through ppi network analysis, we screened 36 hub genes from co-DEGs, including *CCT2*, *ACTR2*, *CLTA* et al. Using the results obtained from the PPI network analysis (Supplementary Figure one to two), we extracted the expression profiles of the hub genes to construct a predictive model. Using LASSO regression, 12 genes were selected with non-zero regression coefficients and value of lambda. min = 0.003690707 (Figures 4A,B). *CCT2* was further used to construct logistic regression prediction models as follows: risk score = (4.0041× ARAF- 1.9746× ACTR2- 5.8043× ATP5F1+ 15.7535× ATP6V1A+ 9.4168× ATP6V1C1- 15.9159× CA10–1.8964× GNG11 + 4.0073× NRXN1- 10.1124× PPFIA2+ 4.2734× PPP1R1B- 11.3482× PPP2CA- 4.1251× RAN+ 2.2023× CCT2). The heatmap indicated the relationship between prediction score and disease, age, and related genes (Figure 4C). The ROC curve indicates that the area under curve (AUC) is 0.9671 and 0.9700 (Figure 4D,E) in the training and validation sets, respectively. In the external validation set (GSE44768, GSE44770, GSE44771, and GSE140829), AUC values were 0.9681, 0.9724, 0.923, and 0.6342 for prefrontal samples, hippocampal samples, cerebellar samples, and whole blood samples, respectively (Figure 4F), indicating that the model has high accuracy in AD prediction.

Additionally, *CCT2* was expressed in all cell types in the human brain (Figure 4G) and significantly downregulated in various brain regions (Figure 4H), indicating that *CCT2* and its related genes are significantly correlated with AD and have broad prospects as a biomarker.

### Transcriptome was combined with micro RNA omics analysis

**FIGURE 5:**
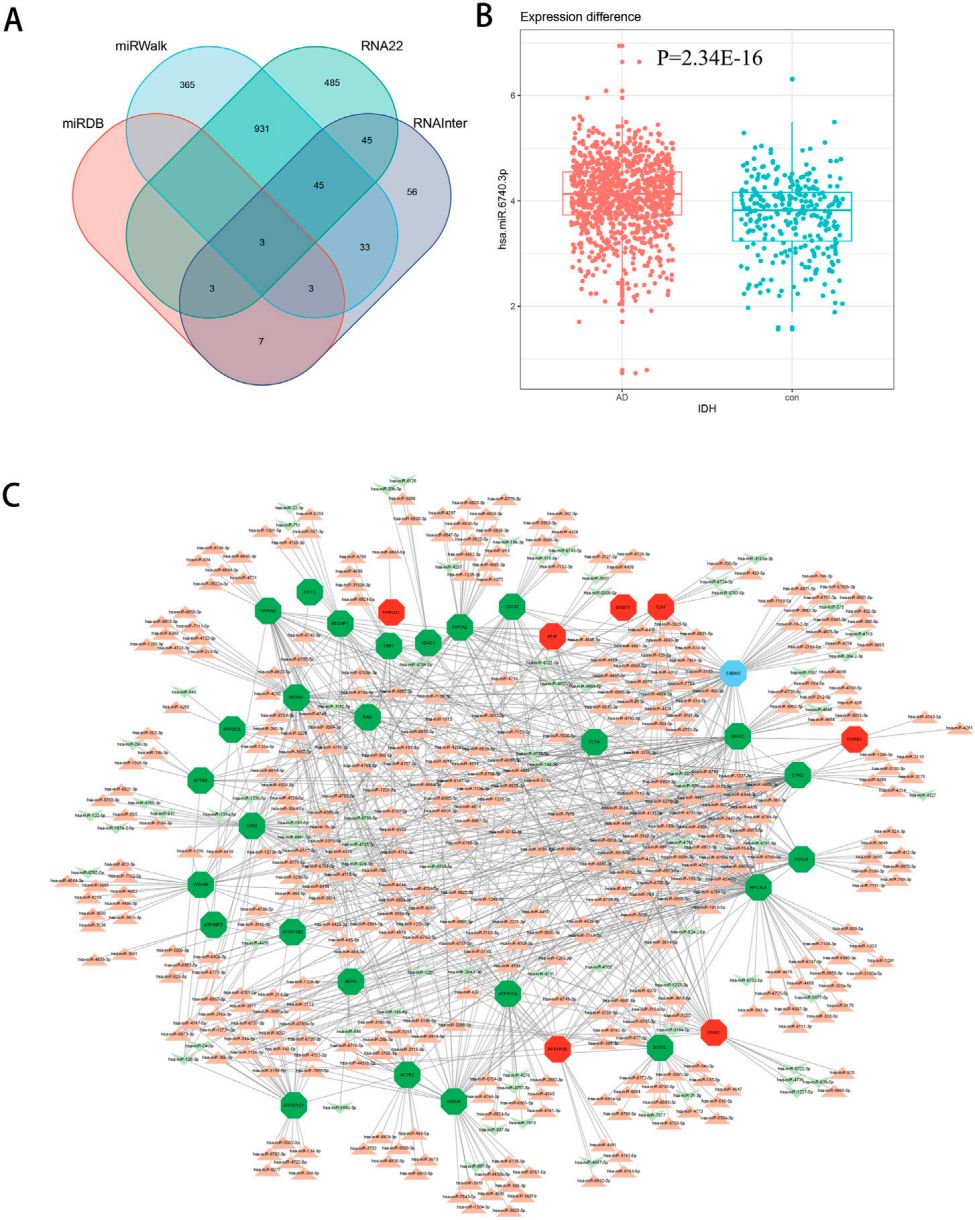
The mRNA-miRNA interaction network. **(A)** Four databases—miRDB, miRWalk, RNA22, and RNAInter—were combined for CCT2-targeting miRNA prediction. **(B)** The boxplot demonstrates the expression level of miR-6740–3p in the serum samples (GSE120584) (logFC = 0.38540845, *p* = 2.34E-16). **(C)** The interaction network presents the hub genes and their corresponding miRNA, with octagon nodes representing genes and other shapes representing miRNA. Upregulation is indicated in red; downregulation is indicated in green.

There are several studies suggesting that miRNA acts on target genes through exosomes and thus affects neurodegenerative diseases (Lydie et al., 2013; Jiang et al., 2019). We used four miRNA databases for joint prediction (Figure 5A), among which three were predicted for *CCT2*, including miR-196b-3p, miR-4778–3p, and miR-6740–3p, where miR-6740–3p was significantly different in blood samples (Figure 5B). Thus, miR-6740–3p may inhibit *CCT2* translation by binding to its transcript, which may be a potential cause of AD. We analyzed all 36 hub genes in the same way, and the mRNA-miRNA interaction network revealed that the majority of the miRNAs corresponding to the downregulated genes in AD were upregulated, confirming that the interaction network had a good predictive value (Figure 5C).

### Small-molecule drug prediction based on the hub genes

*CCT2* may be a novel target for AD therapy, thus, the protein-drug interactions must be predicted. Since macromolecular drugs are difficult to cross the blood-brain barrier, we used the DSingDB database for model gene prediction to identify ten viable small molecule drugs. Additionally, they were sorted and displayed based on the *p*-value (Table 1).

**TABLE 1:** List of drugs recommended for treating AD by targeting CCT2

Name	*p*-value	Chemical formula	Structure
**Amantadine HL60**	3.55E-05	C_10_H_17_N	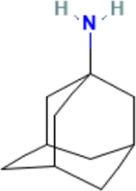
**Flupentixol HL60**	**7.71E-05**	C_23_H_25_F_3_N_2_OS	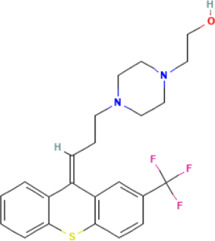
**Flunixin HL60**	4.36E-04	C_14_H_11_F_3_N_2_O_2_	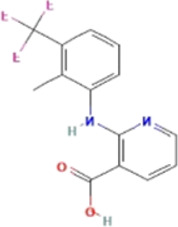
**Cefotiam PC3**	4.88E-04	C_18_H_23_N_9_O_4_S_3_	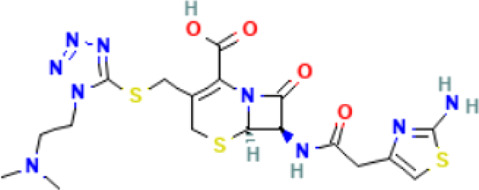
**Primidone PC3**	0.001139	C_12_H_14_N_2_O_2_	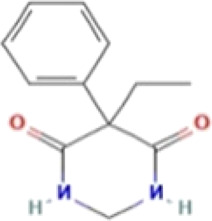
**Clopamide HL60**	0.00128	C_14_H_20_ClN_3_O_3_S	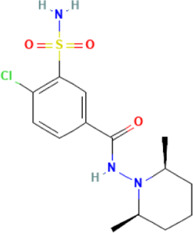
**Hesperidin PC3**	0.001862	C_28_H_34_O_15_	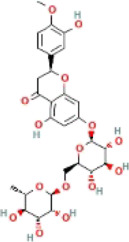
**Reserpine TTD**	0.014852	C_33_H_40_N_2_O_9_	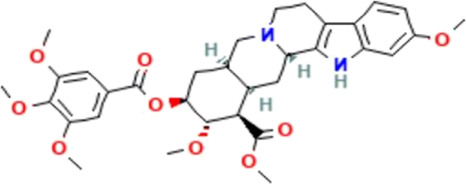
**Enkephalin**	0.016133	C_28_H_37_N_5_O_7_	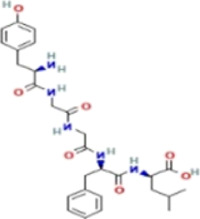
**Caffeic acid PC3**	0.018692	C_9_H_8_O_4_	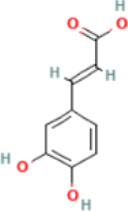

Some studies have shown that *Amantadine* may have a new beneficial effect on axial symptoms of Parkinson’s disease (PD) patients with subthalamic nucleus deep brain stimulation (Chan et al., 2013). Additionally, *Reserpine* is an antihypertensive agent whose action is attributed to its ability to inhibit the vesicle monoamine transporter *VMAT*, thereby reducing the level of bioamine neurotransmitters in synaptic vesicles. One study found that reserpine improves Aβ toxicity in caenorhabditis elegans model of AD (Arya et al., 2009). Similarly, several small-molecule drugs seem to be associated with AD. Elevated enkephalins cause neuronal and behavioral disorders in transgenic mouse models of AD (Meilandt et al., 2008). Caffeic acid slows the development of AD by increasing cognitive function, alleviating brain damage, and inhibiting the AD-induced increase in AChE activity and nitrite production (Wang et al., 2016).

## Discussion

AD has gradually grown to be one of the most significant diseases of this century as a typical neurodegenerative condition affecting the elderly. Over 50 million people worldwide currently suffer from dementia, with AD accounting for 60–80% of all dementia cases (Porsteinsson et al., 2021). Acetylcholinesterase inhibitor (AChEI) is a common drug for the treatment of AD; however, it can only treat its symptoms and have some side effects (Lane et al., 2018). Studies have reported that AD is caused by abnormal folding of Aβ protein, and the neurofibrillary tangles are caused by excessive phosphorylation of Tau (Kozlov et al., 2017), which is accompanied by neuronal apoptosis, which is irreversible. Therefore, the onset of AD is usually irreversible (Ganzer, 2007).

Previous studies have demonstrated the eukaryotic chaperone tailless complex polypeptide one ring complex and its eight subunits can prevent the formation of protein aggregates (Behrends et al., 2006; Noormohammadi et al., 2016). A recently published paper has demonstrated that *CCT2* binds to protein aggregates, recruits autophagosomes to endocytose, and degrades Tau (Ma et al., 2022). Therefore, our study used the transcriptome sequencing dataset of AD prefrontal cells to explore the regulatory mechanism of *CCT2* in AD. We observed that *CCT2* was significantly downregulated in patients with AD, suggesting that the *CCT2* downregulation may be a contributing factor for AD. By considering the intersection of AD-con and *CCT2*-low/high, we identified the common DEGs. Further, using PPI network analysis, 36 hub genes of co-DEGs were screened out, including *CCT2*, *ACTR2*, and *TCIRG1*. Among them, *MYT1L* was observed to induce cell reprogramming into cholinergic neurons and provide a strategy for treating AD (Liang et al., 2018). *ATP6V1A* is involved in AD *via* the synaptic vesicle cycle, phagosome, and oxidative phosphorylation downregulation (Zhou et al., 2021). Additionally, *VDAC1* has been observed to impact AD occurrence by regulating mitochondrial function (Shoshan-Barmatz et al., 2018). This indicates a strong correlation between hub genes and AD occurrence. Since *CCT2* is also closely related to these genes, it may play an important role in the regulation of hub genes, providing a reference for future research.

However, functional enrichment analysis revealed that the genes positively correlated with *CCT2*, selected by Pearson correlation coefficient, were associated with telomerase, Cajal body positioning, mRNA splicing, protein folding, and MAPK activity, indicating that the genes highly associated with *CCT2* are involved in the entire process of transcription and translation. This is also consistent with the mechanism of co-regulation of protein homeostasis by a molecular chaperone and aggregate autophagy reported in a study by Ma et al. (Ma et al., 2022). Meanwhile, the MAPK signaling pathway is believed to function during the early stages of AD (Johnson and Bailey, 2003), and the pathway enrichment analysis revealed that it is related to multiple neurodegenerative diseases and autophagy in animals whereas autophagic deficiency is a widely recognized cause of AD (Reddy and Oliver, 2019; Fang et al., 2019).

Meanwhile, GSEA analysis of the DEGs and GSVA analysis of the *CCT2*-related genes revealed that *CCT2* affects autophagy. GSEA analysis demonstrated that the JAK-STAT pathway, the Notch signaling pathway, angiogenesis, and development and differentiation of glial cells were enriched in AD-con. At the same time, *CCT2*-low/high groups whereas proteasome, animal autophagy, protein breakdown, and neuronal development were not enriched. Several pathways have been reported to correlate with AD occurrence. For example, the JAK-STAT signaling pathway has been reported to play a potential role in the molecular mechanism regulating cellular autophagy (Chen et al., 2021). The Notch pathway is linked to autophagy (Ko et al., 2020), and angiogenesis is also believed to promote AD (Vagnucci and Li, 2003). Additionally, there is evidence that the AD and the CCT2-low expression groups can clear Aβ and Tau by inhibiting proteasomes and autophagy (Bonet-Costa et al., 2016). In contrast, GSVA revealed that the expression level of *CCT2* was negatively associated with neuronal death, suggesting that high *CCT2* expression can inhibit neuronal death. Furthermore, the *CCT2* expression level was significantly and positively correlated with mitophagy, macroautophagy, pexophagy, protein folding, and protein metabolism; however, the R-value for mitophagy was relatively low, possibly since *CCT2* does not directly affect mitophagy. This is consistent with the first report of *CCT2*-mediated aggregate elimination and indicates that *CCT2* can regulate the levels of Aβ and Tau by regulating protein folding and promoting autophagy. Increasing evidence suggests that autophagy plays a role in scavenging abnormal proteins, thus, affecting the clearance of Aβ and Tau when autophagy activation is hampered (Dou et al., 2020). Hence, autophagy dysfunction plays a crucial role in the pathological process of AD (Li et al., 2010).

We also analyzed the correlation between *CCT2* and autophagy-related genes, and top ten autophagy genes with the strongest positive correlation included *BNIP3*, *HSPA8*, and *MAPK8 etc.* Whereas those with the strongest negative correlation included *ATG16L2*, *ATG9B*, and *BAX etc.* This indicates that *CCT2* may affect autophagy by interacting with genes that are highly associated with autophagy. The autophagy gene set correlation analysis revealed that *CCT2* is positively correlated with the initiation stage of autophagy, macroautophagy, and chaperone-mediated autophagy, which also suggested that *CCT2* may affect the occurrence of autophagy *via* some mechanism. We also observed the inhibition of the mammalian target of the rapamycin (mTOR) pathway. Additionally, high expression of mTOR-inhibiting autophagy has been demonstrated in neurodegenerative diseases (Zhu et al., 2019).

During the construction of the clinical prediction model, we used LASSO regression to screen for genes with regression coefficients greater than zero and then combined these genes with *CCT2* to build the logistic model. The model performed well with high AUC values in the prefrontal cortex, visual cortex, and hippocampus; however, it performed poorly in whole blood samples, which could be attributed to the fact that the brain tissue samples were used to construct the model. Some of the genes involved in the model have been observed to be involved in AD, of which *CCT2* is significantly downregulated in AD, *PPP1R1B* can regulate *cAMP response element-binding protein* (*CREB*) phosphorylation, and *CREB* dysfunction is one of the causes of AD (Cho et al., 2015) whereas *NRXN1* is involved in memory recovery in rats by affecting synaptic plasticity (Zhang et al., 2021). The ROC curve revealed that the model had high AUC values in the training, test, and external validation sets; thus, the expression of these genes can be used as a biomarker for AD. Online database analysis also demonstrated that *CCT2* is expressed in various brain cells, with neuronal cells having the highest levels of expression. *CCT2* expression, in contrast, was significantly reduced in various brain regions, including the entorhinal cortex, hippocampus, frontal cortex, and frontal cortex, providing further evidence that low CCT2 expression is one of the mechanisms of AD pathogenesis.

The study also investigated the causes of low *CCT2* expression and observed that gene mutations, DNA methylation, and miRNA may cause changes in gene expression, causing AD (Qin et al., 2020; De Jager et al., 2014; Akhter and Bekris, 2019). Thus, we aimed to explore the miRNA interacting with hub genes, most of which had the opposite expression profile of their target genes in AD. For example, miR-6740–3p, which interacts with *CCT2*, is significantly upregulated in AD (logFC = 0.38540845, *p* = 2.34E-16) and miR-661, which interacts with *HPCAL4*, *NECAP1*, *CLTA*, and *GNAI2*, has been observed to be involved in AD *via* metabolic and stress pathways (Hojati et al., 2021). The miR-501–3p, which interacts with *CADM2*, may impact AD by regulating cell division (Hara et al., 2017); hsa-miR-107, which interacts with *ACTR2*, *AMPH*, and *RAN*, targets Aβ precursor protein (APP) and influences AD (Hébert et al., 2008). This study can assist researchers in screening for appropriate miRNA and validating their biological functions to obtain effective biological results. (The specific pathways by which most miRNAs affect AD are currently unknown, albeit the mRNA-miRNA interaction network serves as a reference for AD diagnosis and treatment. Similarly, the prediction of the last small-molecule drugs provides a point of reference for targeting *CCT2* to treat AD.

## Conclusion

Using bioinformatic analysis, this study used multiple datasets and revealed that the low expression of *CCT2* in AD may be responsible for the inhibition of autophagy in AD. The PPI network was used to screen out potential AD biomarkers with diagnostic value, and the mRNA-miRNA interaction network was constructed to predict the potential miRNA. These findings contribute to our understanding of the pathogenesis of AD and provide new guidelines for the treatment and diagnosis of the disease.

## Data Availability

Publicly available datasets were analyzed in this study. The names of the repository/repositories and accession number(s) can be found in the article/Supplementary Material.
